# New onset of mental disorders, lifestyle changes, and quality of relationships during COVID-19 in italian population

**DOI:** 10.1192/j.eurpsy.2021.286

**Published:** 2021-08-13

**Authors:** D. Caldirola, F. Cuniberti, S. Daccò, A. Defillo, S. Lorusso, G. Perna

**Affiliations:** 1 Mental Health, Humanitas University, Rozzano, Italy; 2 Department Of Clinical Neurosciences, Villa San Benedetto Menni Hospital, Hermanas Hospitalarias, Albese con Cassano, Italy; 3 Health Technology, Medibio Limited, Savage, United States of America

**Keywords:** lifestyle, relationships, COVID-19, Mental disorders

## Abstract

**Introduction:**

The COVID-19 pandemic has been causing relevant public health and psychosocial consequences.

**Objectives:**

To assess the impact of the COVID-19 pandemic on mental health, lifestyle and personal relationships in the Italian general population.

**Methods:**

An online survey spread between May and June 2020 to collect socio-demographic, clinical, lifestyle, relationship, and mental health self-reported information. Mental disorder screening was performed by the Patient Health Questionnaire and PTSD Checklist for DSM-5.

**Results:**

Participants were 2003, 1504 of which (75%) completed the entire questionnaire (1157 females, 77%). Among the completers who have not had any mental disorder before (n=524, 35%), 263 (51.7%) met cut-off scores for psychiatric diagnoses on the self-report psychiatric screeners during the pandemic (i.e., Major Depressive Disorder, 11.3%, with death thoughts in approximately half of the cases; Panic Disorder, 1.1%; Generalized Anxiety Disorder, 13%.3, Obsessive-Compulsive Disorder, 13.2%, Post-Traumatic Stress Disorder, 7.3%; Alcohol Abuse, 5.5%). In line with this, 39% of completers complained of insomnia, while 12% and 10% started using anxiolytics and antidepressants, respectively. Approximately 7-8 % of completers started/increased alcohol and/or nicotine consumption, 33% quitted/decreased physical activity, and 40% declared decreased sexual satisfaction. Approximately 21% and 38% declared worsening in relationship with partner and difficulty in child-caring, respectively.

**Conclusions:**

The COVID-19 pandemic appears to be a risk factor for new onset of mental disorders and worsening in lifestyle and familial relationships in the Italian population. These results should be confirmed by clinical interviews, and may represent a starting point for further monitoring of the medium and long-term consequences of the COVID-19 pandemic.

**Disclosure:**

No significant relationships.

